# CircCCDC91 regulates chicken skeletal muscle development by sponging miR-15 family via activating IGF1-PI3K/AKT signaling pathway

**DOI:** 10.1016/j.psj.2022.101803

**Published:** 2022-02-25

**Authors:** Jing Zhao, Xiyu Zhao, Xiaoxu Shen, Yun Zhang, Yao Zhang, Lin Ye, Diyan Li, Qing Zhu, Huadong Yin

**Affiliations:** ⁎ Farm Animal Genetic Resources Exploration and Innovation Key Laboratory of Sichuan Province, Sichuan Agricultural University, Chengdu, Sichuan 611130, PR China; † College of Management, Sichuan Agricultural University, Chengdu, Sichuan 611130, PR China

**Keywords:** circCCDC91, miR-15 family, IGF1-PI3K/AKT pathway, myoblast, chicken

## Abstract

Circular RNAs (**circRNAs**) has been reported in various tissues of animals and associated with multiple biological processes. From our previous sequencing data, we found a novel circRNA, circCCDC91 which was generated from exon 2 to 8 of the CCDC91 gene. We observed that circCCDC91 was differentially expressed in the chicken breast muscle among 4 different embryonic developmental time points (embryonic day 10 [**E10**], E13, E16, and E19). Therefore, we assumed that circCCDC91 have a potential function in chicken skeletal muscle development. In this study, we firstly verify the annular structure and expression pattern of circCCDC91, and further investigate on whether circCCDC91 could promote chicken skeletal development. Mechanistically, circCCDC91 could absorb miR-15a, miR-15b-5p, and miR-15c-5p to modulate the expression of Insulin receptor substrate1 (**IRS1**), as well as activate insulin-1ike growth factor 1-phosphatidylinositol 3-kinase/AKT (**IGF1-PI3K/AKT**) signaling pathway. In addition, circCCDC91 could rescue skeletal muscle atrophy by activating IGF1-PI3K/AKT pathway. Taken together, the findings in this study revealed that the newly identified circCCDC91 promotes myoblasts proliferation and differentiation, and alleviates skeletal muscle atrophy by directly binding to miR-15 family via activating IGF1-PI3K/AKT signaling pathway in chicken.

## INTRODUCTION

Skeletal muscle accounts for approximately 40 to 60% of the body weight of an adult animal, and plays a crucial role in the overall body metabolism (Yun and Wold, 1997). The growth of the skeletal muscle is closely related to meat production in domestic animals. Myoblasts are indispensable for skeletal muscle development. Myoblasts finally forming myotubes/myofibers through biological processes such as activation, migration, adhesion, membrane reorganization, and nuclear fusion (Lehka and Rędowicz, 2020). Muscle fibers are the functional units of muscle, whose number and size determine the muscle mass (Wigmore and Stickland, 1983). For birds, the number of muscle fibers is determined at birth, and the growth of postnatal muscle mainly depends on the volume of muscle fibers (Buckingham et al., 2003). A decrease in the diameter of muscle fibres is an indication of the occurrence of muscle atrophy, which is usually caused by aging, starvation, prolonged periods of muscle inactivity and some muscle related diseases (Jackman and Kandarian, 2004). The development and atrophy of skeletal muscle are a complex processes, which are regulated by series of biological processes, including signaling pathway, transcription factors, as well as some non-coding RNAs (Yun and Wold, 1997). Among these pathway, IGF1-PI3K/AKT signaling pathway is a key intracellular signaling pathway which regulated the growth and hypertrophy of skeletal muscle (Bodine et al., 2001b; Schiaffino and Mammucari, 2011). A study showed that the pathway prevented muscle atrophy in adult C57BL/6 mice (Tamemoto et al., 1994). IRS1 is an important member of IGF1-PI3K/AKT pathway, it plays a key role in transmitting signals from the Insulin and IGF1 receptors to PI3K/AKT signaling pathway in cells (Schiaffino and Mammucari, 2011). Previous reports indicated that IRS1 regulates skeletal muscle growth, whereas lack of IRS1 causes growth retardation in mice (Tamemoto et al., 1994). In addition, IRS1 has been reported to play key roles in proliferation and differentiation of chicken myoblasts (Li et al., 2019). circRNAs has been considered as a kind of non-coding RNAs with a covalently closed loop that lack 5’caps and 3’tails (Ye et al., 2015). In 1976, researchers initially discovered circRNAs in viruses (Sanger et al., 1976). In recent years, circRNAs have been identified in the transcriptomes of eukaryotes, and their functions are also gradually explored, including acting as a miRNA sponge, participating in regulating the expression of parent linear RNA, coding proteins, and deriving pseudogenes explored (Liu et al., 2018). The emerging evidence revealed that circRNAs participate in many biological processes, including diabetes mellitus, neurological disorders, cardiovascular diseases and cancer (Kristensen et al., 2019). For instance, reports showed that circANKRD36 is closely related to type 2 diabetes mellitus (**T2DM**), and is regarded as a potential biomarker for T2DM (Fang et al., 2018). circPVT1 is involved in the regulation of the head and neck squamous cell carcinoma and aggravating the progression of hepatocellular carcinoma via sponging miR-377 to upregulate TRIM23 (Bu et al., 2020; Verduci et al., 2017). Moreover, circRNAs were also found to be abundantly expressed in the skeletal muscle and play significant role in the development of skeletal muscle (Zhang et al., 2019). Ouyang et al. (2018) firstly identified 3,036 abundantly expressed circRNAs in the chicken embryonic skeletal muscle, and found that circRBFOX2 could accelerate myoblasts proliferation by binding miR-206 and miR-1a-3p. Our research group previously sequenced the breast muscles of broilers and layers at different embryonic developmental time points (E10, E13, E16, and E19), and found that there are many differentially expressed circRNAs between the layer and broiler chicken (Shen et al., 2019). Even though numerous circRNAs have been identified in the chicken skeletal muscles, their specific roles still require further verification.

In our previous sequenced data, we identified 4 circRNAs that were generated by the chicken CCDC91 gene (Supplementary Figure S1A). We also found that a novel and reliable circRNA produced by the exon 2 to 8 of chicken CCDC91 gene (circCCDC91.2-8, we named it as circCCDC91), was highly expressed in the E11, E13, E16, and E19 of the chicken skeletal muscle compared with other circRNAs derived from circCCDC91 (Supplementary Figure S1B–D). Moreover, we further verified circCCDC91 highly expressed in the chicken skeletal muscle using quantitative real-time PCR (**qRT-PCR**) (Supplementary Figure S1E). Therefore, we assumed that circCCDC91 may possess a potential function in the development of chicken skeletal muscle. In this study, we mainly aimed at detecting the potential function and the regulatory mechanism of circCCDC91 in chicken myoblasts. We found circCCDC91 promotes the proliferation and differentiation of chicken myoblasts and rescues muscle atrophy by suppressing the expression of miR-15a, miR-15b-5p, and miR-15c-5p via activating IGF1-PI3K/AKT signaling pathway.

## MATERIALS AND METHODS

### Ethics Standards^1^

#### Tissue Sample Preparation

In this study, tissue samples such as heart, liver, spleen, lung, kidney, breast muscle, leg muscle, brain, intestine, and abdominal fat of 5-day-old Tianfu broiler chickens were obtained and immediately snap-frozen in liquid nitrogen, and then stored at −80℃ freezer until RNA isolation. Each sample had 3 independent replicates.

### RNA Extraction and cDNA Synthesis

Total RNAs were extracted from tissues and cells using Trizol Reagent (Invitrogen, Carlsbad, CA), and the quality and concentration of all the RNA samples were assessed by a Nanodrop 2000 spectrophotometer (Thermo, former Savant, MA). Subsequently, cDNA synthesis was performed using the PrimeScript RT Reagent Kit with the gDNA Eraser Kit (TaKaRa, Tokyo, Japan, for mRNA) and one step miRNA cDNA Synthesis Kit (HaiGene, Haerbin, Heilongjiang, China).

#### Quantitative Real-Time PCR

Synthesized cDNA libraries were diluted with RNase-free water (Tiangen, Beijing, China) at a ratio of 1:3 before qRT-PCR. The qRT-PCR reaction system was 10 µL, including 5 µL of SYBR Green Premix Ex Taq (TaKaRa), 3µL of RNasefree H_2_O (Tiangen), 0.5 µL of forward primer, 0.5 µL of reverse primer, and 1 µL of cDNA. In addition, qRT-PCR reaction conditions were as follows, 95℃ for 3 min, followed by 95℃ for 10 s, the primer-specific annealing temperature for 20 s, and 72℃ for 20 s; these were then repeated 40 times. *GAPDH, β-actin*, and *U6* (for miRNA) were used as the internal control. All relative gene expression levels were collected with CPX Connect Real-Time System (Bio-Rad, Hercules, CA). The 2^−ΔΔCt^ method was used to calculate qRT-PCR data. Each sample was assayed in triplicates.

### Primers

All primers used for qRT-PCR listed in Supplementary Table S1 were designed by Premier Primer 5.0 software (Premier Bio-soft International, Palo Alto, CA) and synthesized by Tsingke (Beijing, China).

### Validation of circCCDC91

To confirm the existence and loop structure of circCCDC91, divergent primers of the across back-splicing junction based on the NCBI reference sequences of CCDC91 (NCBI accession number: XM_015292434) were designed to amplify circCCDC91, chicken myoblasts cDNA was regarded as samples of the PCR reaction as reported (Ouyang et al., 2018; Chen et al., 2019). Subsequently, PCR product of divergent primers was evaluated by Sanger sequencing. RNase R digestion was also used to verify the circular form of circCCDC91. For RNase R treatment, 1 μg of total RNA was incubated for 10 min at 37℃ with 1 unit RNase R, and then deactivated RNase R at 90℃ for 10 min. Finally, the treated RNAs were employed to synthesize cDNA for qRT-PCR. Each sample has three independent replicates.

### Smalls Interfering RNAs and Plasmids Construction

For circCCDC91 overexpression plasmids construction (ov-circCCDC91), the full length of circCCDC91 with cyclization element was cloned into pcDNA3.1-ciR vector (ov-NC) (Geneseed Biotech, Guangzhou, China), and the sequence of circCCDC91 and cyclization elements are listed in Supplementary Table S2. Several small interfering RNAs (si-circCCDC91) and siRNA negative control (si-NC) for circCCDC91 and miR-15 family were designed and synthesized by GenePharma (GenePharma, Shanghai, China) (Supplementary Table S3). For the construction of pmirGLO Dual-Luciferase reporter vector, the wild type (pmirGLO-WT) and mutated sequences (pmirGLO-MT) of circCCDC91 (harbored normal or mutated miR-15a, miR-15b-5p, and miR-15c-5p perfected 5′ seed pairing binding site) were synthesized and cloned into the pmirGLO Dual-Luciferase reporter vector (Sangon Biotech, Shanghai, China).

### Cell Culture and Isolation

The breast muscles of 10-day embryo age (E10) Tianfu broiler chickens were used to isolate myoblasts as described by previous reports (Hongjia et al., 2018). Myoblasts were cultured in Dulbecco's Modified Eagle Medium (**DMEM**; Sigma, St.Louis, MO) with 10% fetal bovine serum (**FBS**; Gibco, Grand Island, NY) and 1% penicillin/streptomycin (Solarbio, Beijing, China) at 37℃ in 5% CO_2_, humidified atmosphere. Chicken fibroblast cell line (DF-1 cells) was also grown in DMEM supplemented with 10% FBS and 1% penicillin/streptomycin.

### Cell Treatment and Transfections

The cells were treated with 10 μM dexamethasone (Solarbio) in DMEM for 24 h, followed cell transfection was performed.

For the proliferation studies, myoblasts were transfected when the cell confluence reached approximately 50%. For differentiation studies, myoblasts were transfected until the confluence approached approximately 90%. Three hours before transfection, cells were cultured in the fresh cell medium without 1% penicillin/streptomycin. Furthermore, the cells were transfected with ov-circCCDC91, ov-NC, si-circCCDC91, si-NC or miR-15a/miR-15b-5p/miR-15c-5p mimics or mimic NC using Lipofectamine 3000 (Invitrogen) and Opti-MEM medium (Gibco) according to the manufacturer's instructions. 96 well plate cells were transfected with 0.1 ug si-RNA, plasmids or miRNA-mimics, 0.2 uL Lipofectamine 3000 and 10 uL Opti-MEM medium. 48-well plate cells were transfected with 0.3 ug si-RNA, plasmids or miRNA-mimics, 0.6 uL Lipofectamine 3000 and 30 uL Opti-MEM medium. 24-well plate cells were transfected with 0.5 ug si-RNA, plasmids or miRNA-mimics, 1 uL Lipofectamine 3000 and 50 uL Opti-MEM medium. 6-well plate cells were transfected with 2 ug si-RNA, plasmids or miRNA-mimics, 4 uL Lipofectamine 3000 and 250 uL Opti-MEM medium. Two/three independent replicates were performed for transfected group per experiments.

### Cell Proliferation Assay

Cell counting kit-8 (**CCK-8**) assay and 5-Ethynyl-2’-Deoxyuridine (**EdU**) assay were used to assess cell proliferation activity. For the CCK-8 assay, chicken myoblasts were plated into 96-well plates. After 12, 24, 36, and 48 h of transfection, 10 μL CCK-8 reagent were added to the cells (Bestbio, Shanghai, China) and the cells were incubated for 1 h at 37℃, subsequently, the absorbance at 450 nm was measured using a Microplate Reader (Thermo). Each treatment group had eight independent replicates. Furthermore, we performed EdU assays, the chicken myoblasts were seeded in 96-well plates, and the proliferating cells were detected using Cell-LightTM EdU Apollo 567 In Vitro Kit (Ribobio, Guangzhou, China). Thereafter, the cells were incubated with 50 µM EdU for 3h at 37℃. Subsequently, myoblasts were fixed with 4% paraformaldehyde (Beyotime, Shanghai, China, P0099) for 30 min and permeabilized with 0.5% Triton X-100 for 20 min. We further stained the cells with Apollo and Hoechst 33,342 for 10 min. Then the EdU-stained cells images were observed and collected by a fluorescence microscope. The number of EdU-stained cells was counted using the Image-Pro Plus 6.0 software (Media Cybernetics, Bethesda, MD). Each treatment group had 6 independent replicates.

### Flow Cytometry Analysis of the Cell Cycle

Chicken myoblasts were plated in 6-well plates and collected after 48 h transfection. Then, cells were fixed with 70% ethanol overnight at 4℃. Subsequently, the fixed myoblasts were incubated with 500 µL PI/RNase Staining Buffer Solution (BD Biosciences, Franklin Lakes, NJ) for 15 min at 37℃. The cell cycles were analyzed by flow cytometry (BD Biosciences). Each treatment group was assayed in triplicates.

### Immunofluorescence

We further performed immunofluorescence analysis. The chicken myoblasts were seeded in 24-well plates and transfected at the stage of 90% confluence. After 72 h of transfection, myoblasts were fixed with 4% paraformaldehyde (Beyotime) for 30 min and washed 3 times with PBS. Subsequently, cells were permeabilized for 20 min using 0.1% Triton X-100 (Coolaber, Beijing, China) and blocked for 30 min with goat serum (Beyotime). The cells were incubated at 4℃ overnight with diluted primary antibody-MyHC (Santa Cruz Biotechnology, Dallas, TX, 1:300, SC-32732). After the overnight incubation, the cells were washed three times with PBS. Then, Rhodamine (TRITC) AffiniPure Goat Anti-Mouse Immunoglobulin G (**IgG**) (ZenBio, Chengdu, China, 1: 1,000, 511102) was added to the plate and further incubated at 37℃ for 1 h. Thereafter, 4’, 6-diamidino-2-phenylindole (DAPI, Beyotime, 1:50) was added to stain the cell nuclei. Finally, the images were randomly captured with a fluorescence microscope (Olympus, Japan). The area of the stained myotubes was calculated by Image-Pro Plus 6.0 software. Each treatment group has three independent replicates.

### Western Blot

Chicken myoblasts were seeded in 6-well plates. After transfection for 72 h, the total proteins were collected using Total Protein Tissue or Cell Total Protein Extraction kit (Solarbio) and evaluated using a bicinchoninic acid (**BCA**) protein assay kit (BestBio, Shanghai, China). We further separated the proteins by SDS-polyacrylamide gel electrophoresis (**SDS-PAGE**) and transferred it to 0.45 um polyvinylidene fluoride (**PVDF**) membranes (Millipore Corporation, Billerica, MA). Subsequently, the PVDF membranes were blocked with Quickblock closed solution (Beyotime) at room temperature for 1 h and incubated overnight with primary antibodies specific for anti-AKT (Cell Signaling Technology, Boston, MA, 1:1,000, 9272S), anti-p-AKT (Cell Signaling Technology, 1:1,000, 9271T), anti-IRS1 (Abcam, London, UK; 1:1,000, ab46800), anti-MyoG (Biorbyt, Cambridge, UK; 1:1,000, orb6492), anti-MyHC (Santa Cruz Biotechnology, 1:1,000, SC-32732), anti-CDK2 (Abclonal, Wuhan, China; 1:1,000, A0294), anti-Atrogin-1 (Abclonal, 1:1,000, A3193), or anti-GAPDH (Zenbio, 1:2,000, 250133). After the overnight incubation, PVDF membranes were washed three times with washing buffer (Beyotime) and then was further incubated with horseradish peroxidase-conjugated secondary antibodies (Zenbio, 1:1,000, 511103) at room temperature for 1 h. Thereafter, the PVDF membranes were washed again using washing buffer (Beyotime). Finally, the protein bands were enhanced by the enhanced chemiluminescence (**ECL**) kit (Beyotime) and then observed with the Image lab software (National Health Institute, Bethesda, MD). *GAPDH* was used as the internal reference for the experiment. The relative protein levels were measured using Image J software. Each treatment group had 2 or 3 independent replicates.

### Target miRNA and Gene Prediction and Luciferase Reporter Assay

Target miRNA prediction was performed by RNAhybrid software (https://bibiserv.cebitec.uni-bielefeld.de/rnahybrid/). The target genes prediction of miRNA was preformed using TargetScan (http://www.targetscan.org/vert_71/). To further investigate the binding sites of miR-15a, miR-15b-5p, and miR-15c-5p on circCCDC91, DF-1 cells were seeded in 48-well plates and then co-transfected with pmirGLO-WT or pmirGLO-MT and miR-15a/miR-15b-5p/miR-15c-5p mimics or mimic NC. After transfection for 48 h, Dual-GLO Luciferase Assay System Kit (Promega, Madison, WI) was used to detect the luminescent values of firefly and Renilla luciferase by a Fluorescence/ Multi-Detection Microplate Reader (Biotek, Winooski, VT).

### Statistical Analysis

All results were presented as a mean ± standard error (**SEM**). For 2 group comparison analysis, statistical significance of differences between means was analyzed by unpaired Student's *t* test. For multiple comparison analysis, the data were analyzed by one-way ANOVA analysis using SPSS 19.0 (SPSS Inc., Chicago, IL), and the significant level were set at *P < 0.05, **P < 0.01, and ^a,b^*P* < 0.05.

## RESULTS AND DISCUSSION

### Confirmation of Interference and Overexpression Efficiency of circCCDC91

**Figure 1:**
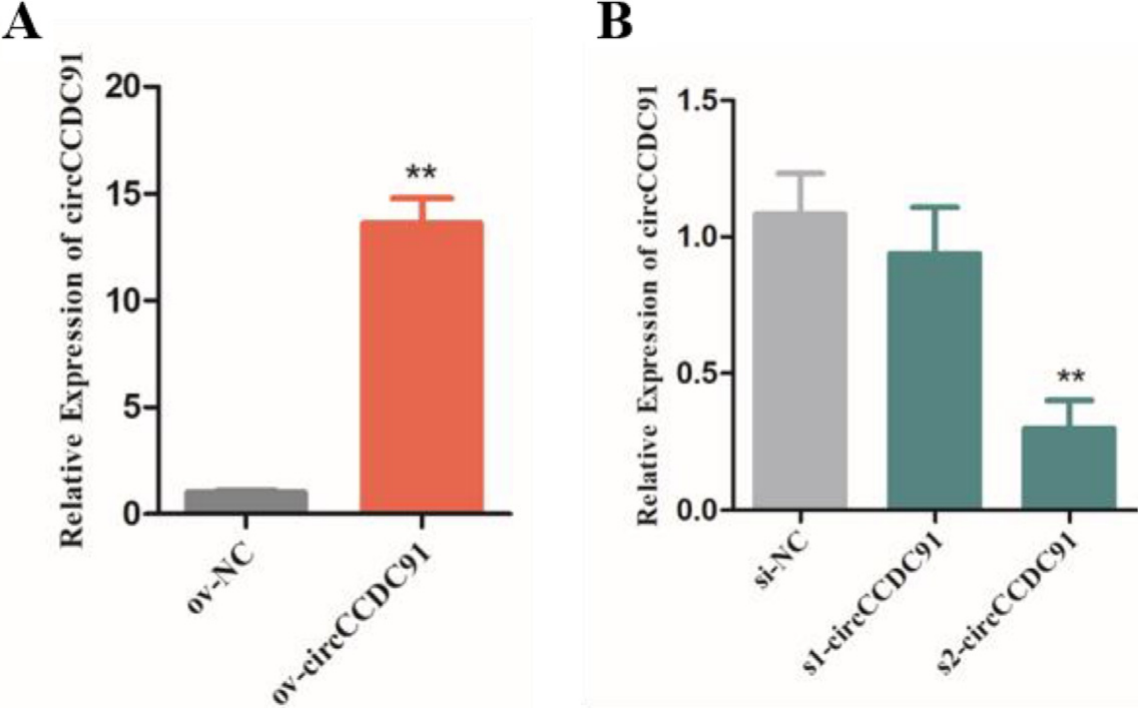
Confirmation of interference and overexpression efficiency of circCCDC91. (A) The expression level of circCCDC91 after transfected with ov-circCCDC91 and ov-NC in chicken myoblasts. (B) The expression level of circCCDC91 after transfected with two siRNAs and si-NC in chicken myoblasts. Data are represented mean ± SEM. Each transfection group has three independent replicates. * *P* < 0.05; ** *P* < 0.01.

Previous reports have showed that the function of circRNAs was researched through specific interference and exogenous increases of its expression level (Chen et al., 2019; Peng et al., 2019). In our study, to investigate the role of circCCDC91 on the development of chicken skeletal muscle, we transfected chicken myoblasts with ov-circCCDC91, ov-NC, si-circCCDC91, and si-NC to alter the expression of circCCDC91, and after 24 h of transfection, the expression level of circCCDC91 was detected by qRT-PCR, As expected, the expression of circCCDC91 significantly upregulated in ov-circCCDC91 transfected group compared with ov-NC (Figure 1A). In addition, between the two si-RNAs, s2-circCCDC91 had the highest interference efficiency on the expression level of circCCDC91 and was selected for the further experiments, which was named as si-circCCDC91 in subsequent experiments (Figure 1B).

### CircCCDC91 Promotes the Proliferation of Chicken Myoblasts

**Figure 2:**
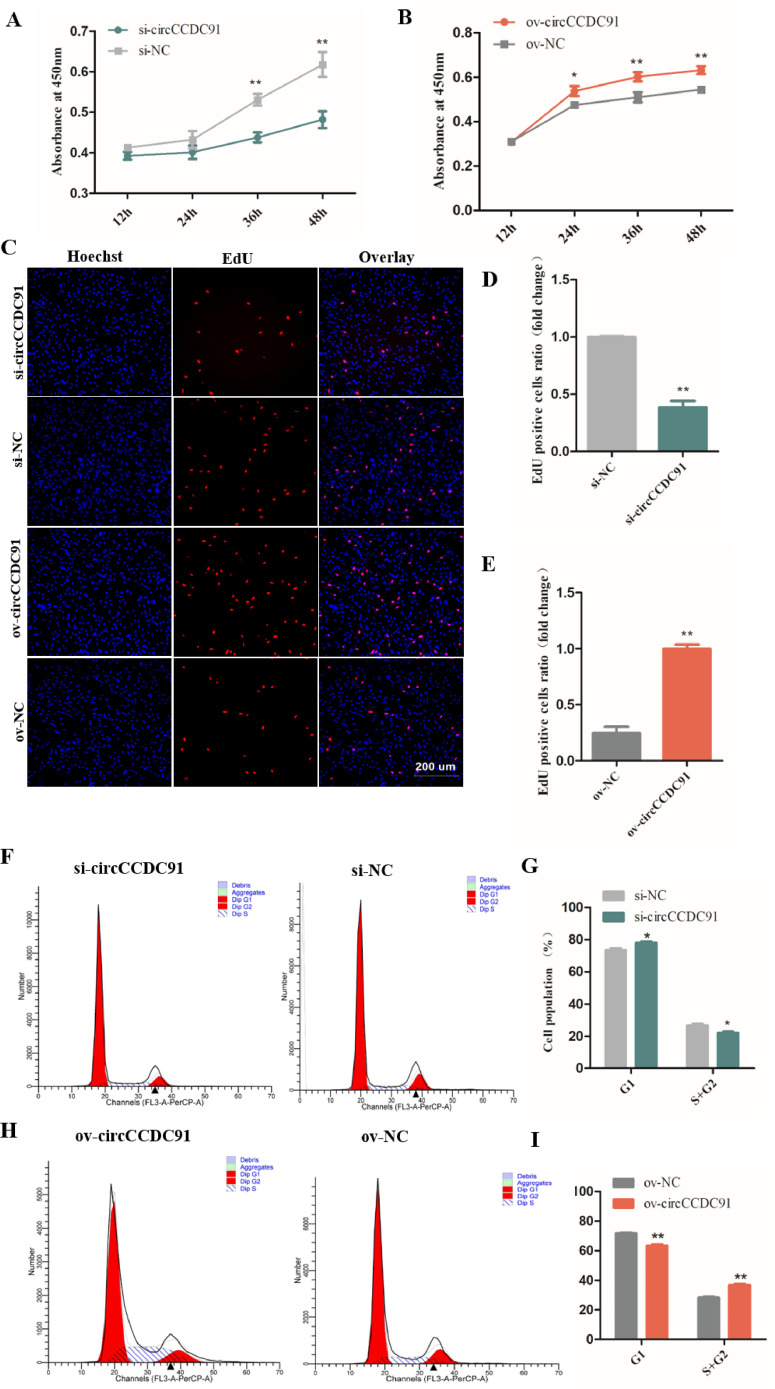
CircCCDC91 promotes the proliferation of chicken myoblasts. (A) CCK-8 assays for myoblasts transfected with si-circCCDC91 and si-NC. (B) CCK-8 assays for myoblasts transfected with overexpression vector of circCCDC91 and ov-NC. (C) EdU assays for myoblasts transfected with si-circCCDC91 and ov-circCCDC91. EdU (red) fluorescence indicates proliferation. Hoechst (blue) fluorescence indicates nuclei. (D) The number of EdU-stained cells was counted after myoblasts transfected with si-circCCDC91 and si-NC. (E) The number of EdU-stained cells was counted after myoblasts transfected with ov-circCCDC91 and ov-NC. (F) Cell cycle analysis for myoblasts after knockdown of circCCDC91. (G) The statistical results of cell cycle analysis for myoblasts after knockdown of circCCDC91. (H) Cell cycle analysis for myoblasts after overexpression of circCCDC91. (I) The statistical results of cell cycle analysis for myoblasts after overexpression of circCCDC91. Data are represented mean ± SEM. For CCK-8 assay, each transfection group has eight independent replicates. For EdU assay, each transfection group has six independent replicates. For the other, each transfection group has three independent replicates. * *P* < 0.05; ** *P* < 0.01.

In recent years, circRNAs have been extensively discovered and identified in chicken skeletal muscle and play an important role in the skeletal muscle development (Ouyang et al., 2018). In the present study, we assessed the effect of circCCDC91 on proliferation of chicken myoblasts by CCK-8, EdU, and cell cycle analyses after transfection with si-circCCDC91, si-NC, ov-circCCDC91, and ov-NC. The results obtained from CCK-8 assay showed that the proliferation vitality of myoblasts significantly decreased after si-circCCDC91 transfection (Figure 2A). Conversely, the ov-circCCDC91 transfected groups had a higher cell proliferation activity, compared with the ov-NC transfected groups (Figure 2B). In addition, the EdU assay results indicated that knockdown of circCCDC91 notably reduced the numbers of EdU stained cells (Figures 2C and 2D). In contrast, overexpression of circCCDC91 markedly increased the cell proliferation rate (Figures 2C and 2E). Finally, cell cycle analysis results showed that the cell population at the S and G2 phase was decreased and the cell population at the G1 phase was increased in the si-circCCDC91 transfected groups (Figures 2F and 2G). On the contrary, exogenous circCCDC91 significantly increases the number of cells in the S and G2 phase and decreased the number of cells in the G1 phase (Figures 2H and 2I). Together, these results suggested that circCCDC91 promotes chicken myoblasts proliferation. Our results are consistent with the findings by Ouyang et al. (2018) who reported that chicken circRBFOX2.2-3 and circRBFOX2.2-4 facilitated myoblasts proliferation. In addition, circRNA circHIPK3 expressed in favor of chicken myoblasts proliferation (Chen et al., 2019). Our research group recently reported that a novel circRNA circTMTC1 inhibits chicken skeletal muscle satellite cell proliferation (Shen et al., 2019). However, in this study, circCCDC91 has shown an opposite effect on skeletal muscle development.

### CircCCDC91 Promotes the Differentiation of Chicken Myoblasts

**Figure 3:**
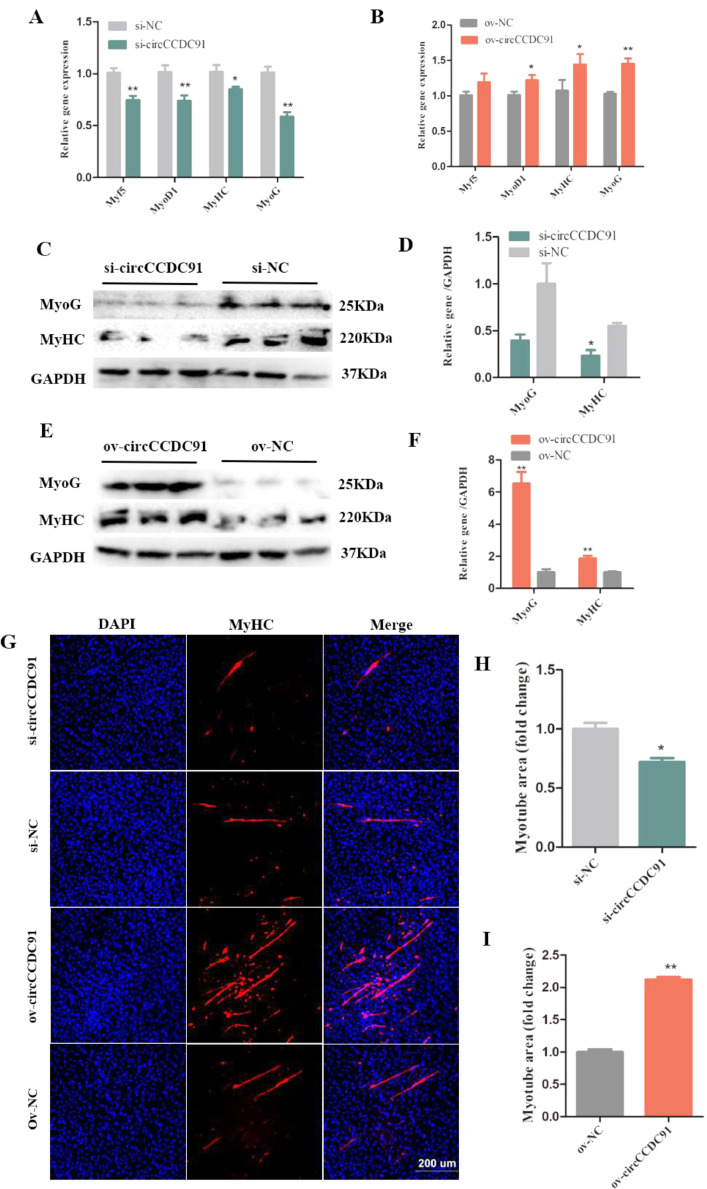
CircCCDC91 promotes the differentiation of chicken myoblasts. (A) The mRNA level of Myf5, MyoD1, MyHC, and MyoG was detected by qRT-PCR after myoblasts transfected with si-circCCDC91 and si-NC. (B) The mRNA level of Myf5, MyoD1, MyHC, and MyoG was detected by qRT-PCR after myoblasts transfected with ov-circCCDC91 and ov-NC. (C) The western blot results of MyoG and MyHC protein expression after circCCDC91 knockdown. (D) The quantification of MyoG and MyHC protein expression after circCCDC91 knockdown. (E) The western blot results of MyoG and MyHC protein expression after circCCDC91 overexpression. (F) The quantification of MyoG and MyHC protein expression after circCCDC91 overexpression. (G) Immunofluorescence analysis of MyHC after knockdown or overexpression of circCCDC91. (H) The percentage of myotube area to total cell area is calculated after transfection with si-circCCDC91 and si-NC. (I) The percentage of myotube area to total cell area is calculated after transfection with ov-circCCDC91 and ov-NC. GAPDH serves as the internal reference for the experiment. Data are represented mean ± SEM. Each transfection group has three independent replicates. * *P* < 0.05; ** *P* < 0.01.

Myogenic regulatory factors (**MRFs**) are important transcription factors family for the process of skeletal muscle development. MRFs activate the formation of muscle fiber and regulate the transcription of specific genes associated with skeletal muscle. MRFs mainly consists of myogenic differentiation 1 (**MyoD1**), myogenin (**MyoG**), and myogenic factor 5 (**Myf5**) (Rudnicki et al., 1994; Berkes and Tapscott, 2005; Cao et al., 2006). In addition, Myosin Heavy Chains (**MyHC**) is a differentiation marker gene of muscle, and it could form the backbone of the sarcomere thick filaments (Tajsharghi and Oldfors, 2013). In this study, to detect the potential effect of circCCDC91 on chicken myoblasts differentiation, the expressions of Myf5, MyoD1, MyoG, and MyHC were determined by qRT-PCR after transfection with si-circCCDC91, si-NC, ov-circCCDC91, and ov-NC. The results showed that the expression of Myf5, MyoD1, MyoG, and MyHC was significantly reduced in the si-circCCDC91 transfected group (Figure 3A), while in the ov-circCCDC91 transfected group, the expression of MyoD1, MyoG and MyHC significantly promoted (Figure 3B). In addition, we analyzed the protein levels of MyoG and MyHC by western blotting, and the results were similar to that of qRT-PCR, the protein level of MyoG and MyHC were decreased in the si-circCCDC91 transfected group (Figures 3C and 3D). On the contrary, upregulation of circCCDC91 obviously improved the protein levels of MyoG and MyHC (Figures 3E and 3F). Moreover, as shown in Figures 3G–3I, we found the myotube areas in the si-circCCDC91 transfected group were lower than that of the si-NC transfected group, whereas overexpression of circCCDC91 obviously accelerated the formation of myotube. Taken together, these results suggested that circCCDC91 promotes the differentiation of chicken myoblasts. Likewise, the effect of circCCDC91 on chicken muscle differentiation was consistent with circSVIL and circHIPK3, both of which could accelerate chicken myoblast differentiation (Hongjia et al., 2018; Chen et al., 2019). However, circCCDC91 and circTMTC1 play different roles in skeletal muscle development. For instance, circCCDC91 promotes chicken skeletal muscle differentiation, whereas circTMTC1 represses chicken skeletal muscle differentiation (Shen et al., 2019).

### CircCCDC91 Acts as a Sponge for miR-15a, miR-15b-5p, and miR-15c-5p to Regulate IRS1 Expression

**Figure 4:**
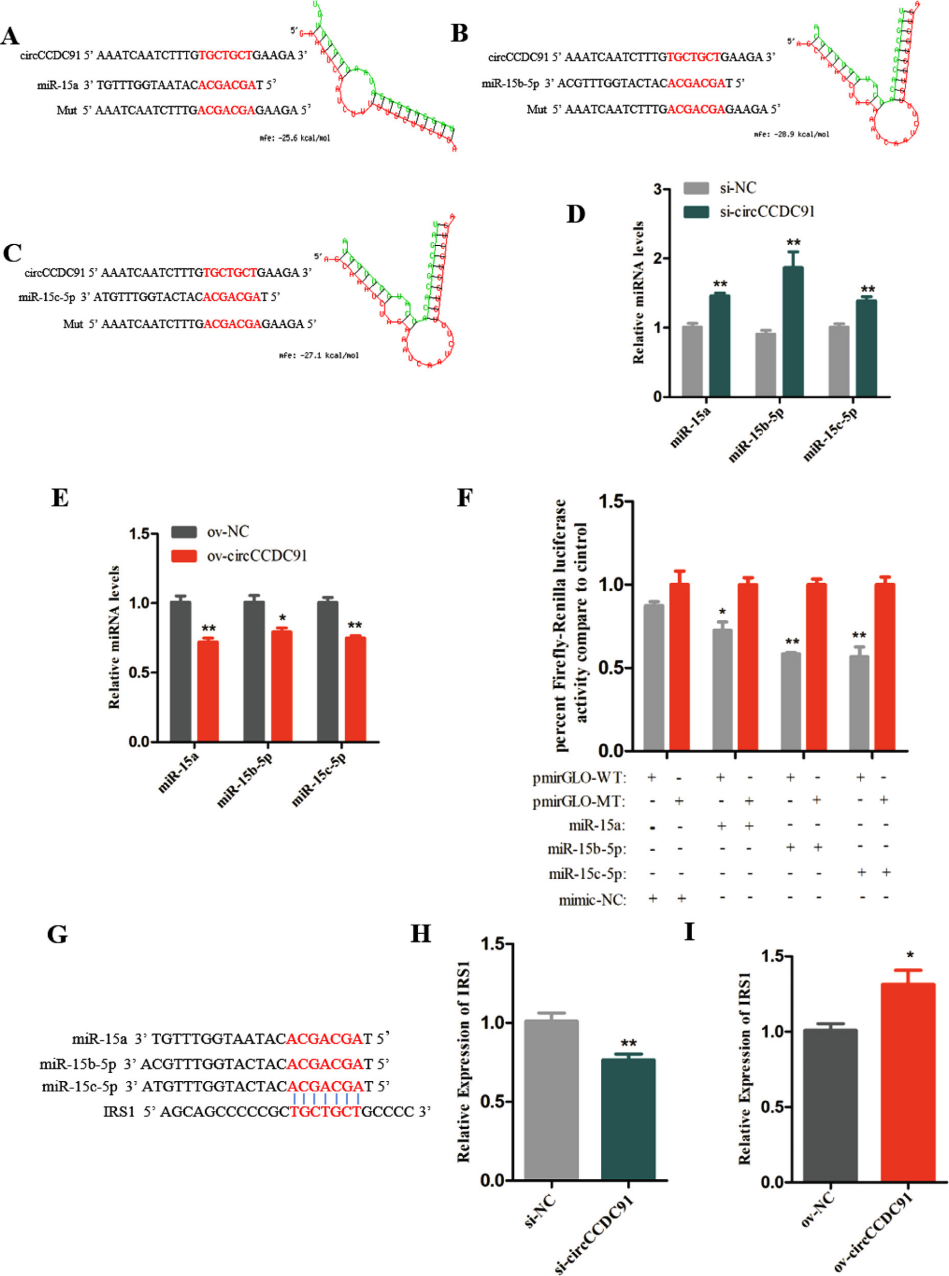
CircCCDC91 acts as a sponge for miR-15a, miR-15b-5p, and miR-15c-5p to regulate IRS1 expression. (A) The potential binding sites of miR-15a in circCCDC91. (B) The potential binding sites of miR-15b-5p in circCCDC91. (C) The potential binding sites of miR-15c-5p in circCCDC91. The seed sequences and mutant sequences are highlighted in red. (D) The expression level of miR-15a, miR-15b-5p, and miR-15c-5p was detected by qRT-PCR after myoblasts transfected with si-circCCDC91 and si-NC. (E) The expression level of miR-15a, miR-15b-5p, and miR-15c-5p was detected by qRT-PCR after myoblasts transfected with ov-circCCDC91 and ov-NC. (F) The dual-luciferase reporter assay was performed in DF-1 cells that co-transfected with pmirGLO-circCCDC91-WT or pmirGLO-circCCDC91-MT and miR-15a/miR-15b-5p/miR-15c-5p mimic or mimic NC. (G) The binding sites analysis in IRS1 3’ UTR. The seed sequences are highlighted in red. (H) The mRNA level of IRS1 was detected by qRT-PCR after myoblasts transfected with si-circCCDC91 and si-NC. (I) The mRNA level of IRS1 was detected by qRT-PCR after myoblasts transfected with ov-circCCDC91 and ov-NC. Data are represented mean ± SEM. Each transfection group has three independent replicates. * *P* < 0.05; ** *P* < 0.01.

Among circRNAs, ceRNAs has been the most generally studied, because circRNA competitively absorbed miRNAs to relieve the inhibitory effect of miRNA on target genes (Kristensen et al., 2019). For example, circFGFR4 acted as a competing endogenous RNA for miR-107 to regulate the expression of Wnt3a (Li et al., 2018). CircSNX29 sponged miR-744 to improve the expression of Wnt5a and CaMKIId (Peng et al., 2019). In our study, we predicted the potential combination sites between circCCDC91 and miRNAs by RNAhybrid, and the results showed that circCCDC91 had binding sites for miR-15a, miR-15b-5p, and miR-15c-5p, respectively (Figures 4A4C). It has been reported that miR-15 family played a regulatory role in mitosis of cardiomyocytes, cardiomyocyte survival, and cardiac repair (Porrello et al., 2011). Besides, miR-15 family has also been reported to be involved in the migration of trophoblast and endothelial cells (Finnerty et al., 2010; Gagnon et al., 2019). Importantly, miR-15 family is also a key regulator of skeletal muscle development (Cimmino et al., 2005). Therefore, to further identify circCCDC91 sponges miR-15 family, we measured the RNA levels of miR-15a, miR-15b-5p, and miR-15c-5p after transfection with si-circCCDC91, si-NC, ov-circCCDC91, and ov-NC in myoblasts. We found knockdown of circCCDC91 distinctly accelerated the expression of miR-15a, miR-15b-5p, and miR-15c-5p (Figure 4D). Conversely, exogenous circCCDC91 significantly reduced miR-15a, miR-15b-5p, and miR-15c-5p expression (Figure 4E). Meanwhile, luciferase assay revealed that miR-15a, miR-15b-5p, and miR-15c-5p significantly reduced the relative luciferase activity of the pmirGLO-WT plasmid compared with the pmirGLO-MT plasmid (Figure 4F). In summary, these results suggested that circCCDC91 could act as a molecular sponge for miR-15a, miR-15b-5p, and miR-15c-5p.

In addition, we predicted the target genes of miR-15 family by TargetScan online software tools, and the results showed that IRS1 possessed conserved target sites for miR-15a, miR-15b-5p, and miR-15c-5p (Figure 4G). IRS1 played a significant role in skeletal muscle development, whereas its deficiency was reported to cause growth retardation in mice (Tamemoto et al., 1994). Subsequently, we found that the mRNA level of IRS1 in circCCDC91 knockdown cells was dramatically lower than that in control, while the expression of IRS1 in the ov-circCCDC91 transfection group was higher than that in the control group (Figures 4 H and 4I). Altogether, the above results suggested that circCCDC91 acts as a sponge for miR-15a, miR-15b-5p, and miR-15c-5p to regulate IRS1 expression.

### CircCCDC91 Can Activate IGF1-PI3K/AKT Signaling Pathway

**Figure 5:**
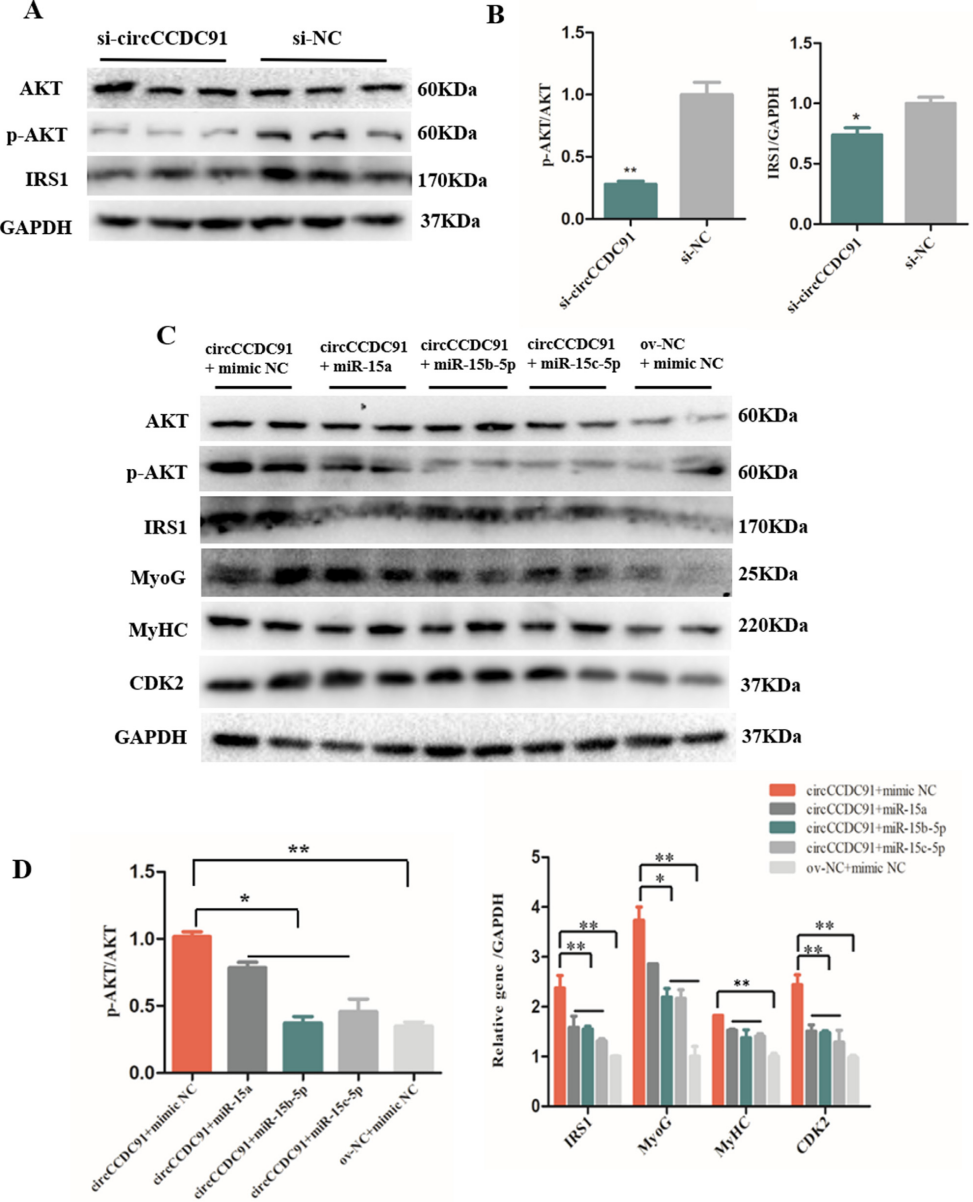
CircCCDC91 can activate IGF1-PI3K/AKT signaling pathway (A) The western blot results of AKT, p-AKT, and IRS1 protein expression after circCCDC91 knockdown. (B) The quantification of p-AKT and IRS1 protein expression after circCCDC91 knockdown. (C) The western blot results of AKT, p-AKT, IRS1, MyoG, MyHC, and CDK2 protein expression after myoblasts co-transfected with ov-circCCDC91 or ov-NC and miR-15a/miR-15b-5p/miR-15c-5p mimic or mimic NC. (D) The quantification of AKT, p-AKT, IRS1, MyoG, MyHC and CDK2 protein expression after myoblasts co-transfected with ov-circCCDC91 or ov-NC and miR-15a/miR-15b-5p/miR-15c-5p mimic or mimic NC. GAPDH serves as the internal reference for the experiment. Data are represented mean ± SEM. For si-circCCDC91 and si-NC transfection group, each group has three independent replicates. For circCCDC91 and miR-15 co-transfected group, each group has two independent replicates. * *P* < 0.05; ** *P* < 0.01.

IRS1 is a signaling adapter protein that transmit signals from IGF1 receptors to intracellular signaling pathway PI3K/AKT. IGF1-PI3K/AKT pathway is a key intracellular signaling pathway which regulates many biological progresses. Interestingly, IGF1-PI3K/AKT pathway was reported to play key roles in for skeletal muscle hypertrophy, atrophy and myogenesis. For instance, IGFBP-5 was reported to promote myogenesis through the IGFI-PI3K-AKT signaling pathway in C2C12 myoblast (Ren et al., 2008). MiR-18a acts as a positive regulator of C2C12 atrophy by inhibiting IGF1-PI3K/AKT signaling pathway (Liu et al., 2017). To determine whether circCCDC91 could regulate IGF1-PI3K/AKT signaling pathway by suppressing the expression of miR-15a, miR-15b-5p, and miR-15c-5p, we transfected myoblasts with si-circCCDC91 and si-NC, whereas ov-circCCDC91 or ov-NC and miR-15a/miR-15b-5p/miR-15c-5p mimic or mimic NC were co-transfected into myoblasts. After the transfection, western blots were performed and the results showed that the protein levels of IRS1 and p-AKT was significantly decreased by knockdown of circCCDC91 (Figures 5A and 5B). Besides, the level of IRS1 and p-AKT were significantly upregulated in the ov-circCCDC91 and mimic NC co-transfected group compared with the ov-circCCDC91 and miR-15a/miR-15b-5p/miR-15c-5p mimic co-transfected group and the ov-NC and mimic NC co-transfected group (Figures 5C and 5D). In addition, we evaluated the protein levels of the differentiated marker gene and the proliferation marker gene CDK2, the results were similar to the protein levels of IRS1 and p-AKT after co-transfected with ov-circCCDC91 or ov-NC and miR-15a/miR-15b-5p/miR-15c-5p mimic or mimic NC into myoblasts (Figures 5C and 5D). Together, these results suggested that circCCDC91 upregulates IRS1 expression via sponging miR-15a, miR-15b-5p, and miR-15c-5p to activate IGF1-PI3K/AKT signaling pathway, further regulates the proliferation and differentiation of chicken myoblast.

### CircCCDC91 Rescues Skeletal Muscle Atrophy

**Figure 6:**
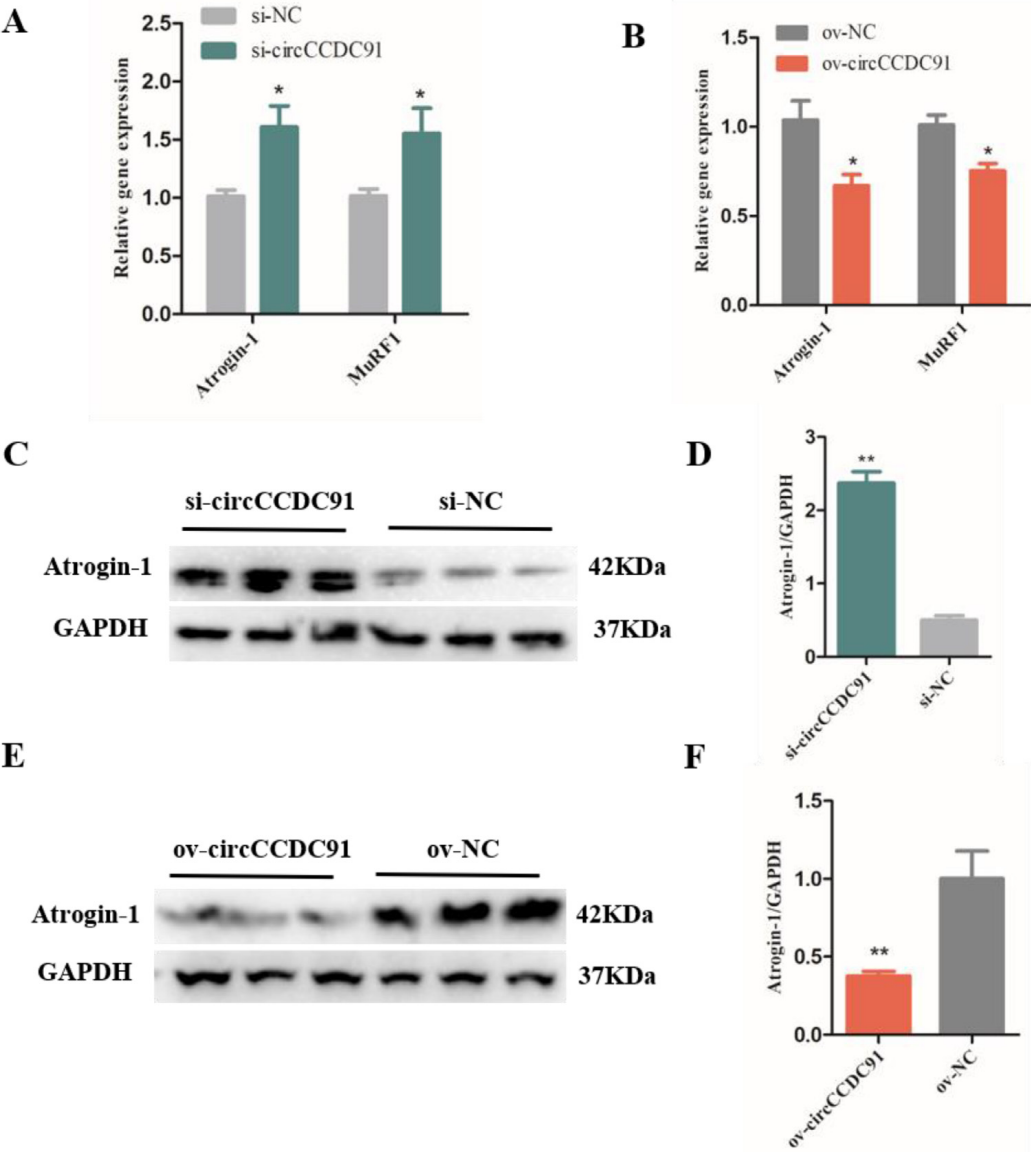
CircCCDC91 rescues skeletal muscle atrophy. (A) The mRNA level of Atrogin-1 and MuRF1 was detected by qRT-PCR in myoblasts after transfected with si-circCCDC91 and si-NC. (B) The mRNA level of Atrogin-1 and MuRF1 was detected by qRT-PCR in myoblasts after transfected with ov-circCCDC91 and ov-NC. (C) The western blot results of Atrogin-1 protein expression after circCCDC91 knockdown. (D) The quantification of Atrogin-1 protein expression after circCCDC91 knockdown. (E) The western blot results of Atrogin-1 protein expression after circCCDC91 overexpression. (F) The quantification of Atrogin-1 protein expression after circCCDC91 overexpression. GAPDH serves as the internal reference for the experiment. Data are represented mean ± SEM. Each transfection group has three independent replicates. * *P* < 0.05; ** *P* < 0.01.

Muscle atrophy could be caused by starvation, prolonged periods of muscle inactivity and some muscle related diseases, it is characterized by a reduction in protein content, fiber diameter, force production, and fatigue resistance reduction (Jackman and Kandarian, 2004). Previous studies have identified 2 important genes that are upregulated in multiple models of skeletal muscle atrophy, and are specifically expressed in skeletal and cardiac muscle. Hence, they were regarded as markers of atrophy process (Glass, 2003). The 2 genes are called muscle atrophy F-box (MAFbx/Atrogin-1) and muscle RING finger-1 (**MuRF1**) (Glass, 2003; Foletta et al., 2011). Atrogin-1 and MuRF1 are number of the ubiquitin proteasome pathway (**UPP**), and the UPP participated in intracellular protein degradation in skeletal muscle (Lecker et al., 1999). In this study, we verified the role of circCCDC91 in the chicken skeletal muscle atrophy by evaluating the expression of Atrogin-1 and MuRF1 after knockdown or overexpression of the circCCDC91 mRNA levels. We found that knockdown of circCCDC91 facilitated Atrogin-1 and MuRF1 expression (Figure 6A), while circCCDC91 overexpression inhibited the expression of these 2 muscular atrophy-related genes (Figure 6B). We further performed western blot analysis to determine the protein levels of Atrogin-1, and the results showed that Atrogin-1 protein level of the si-circCCDC91 transfected group was obviously higher than that of the negative control group (Figures 6C and 6D), while the protein level of Atrogin-1 was significantly reduced in myoblasts transfected with ov-circCCDC91 compared to the ov-NC transfected group (Figures 6E and 6F).

**Figure 7:**
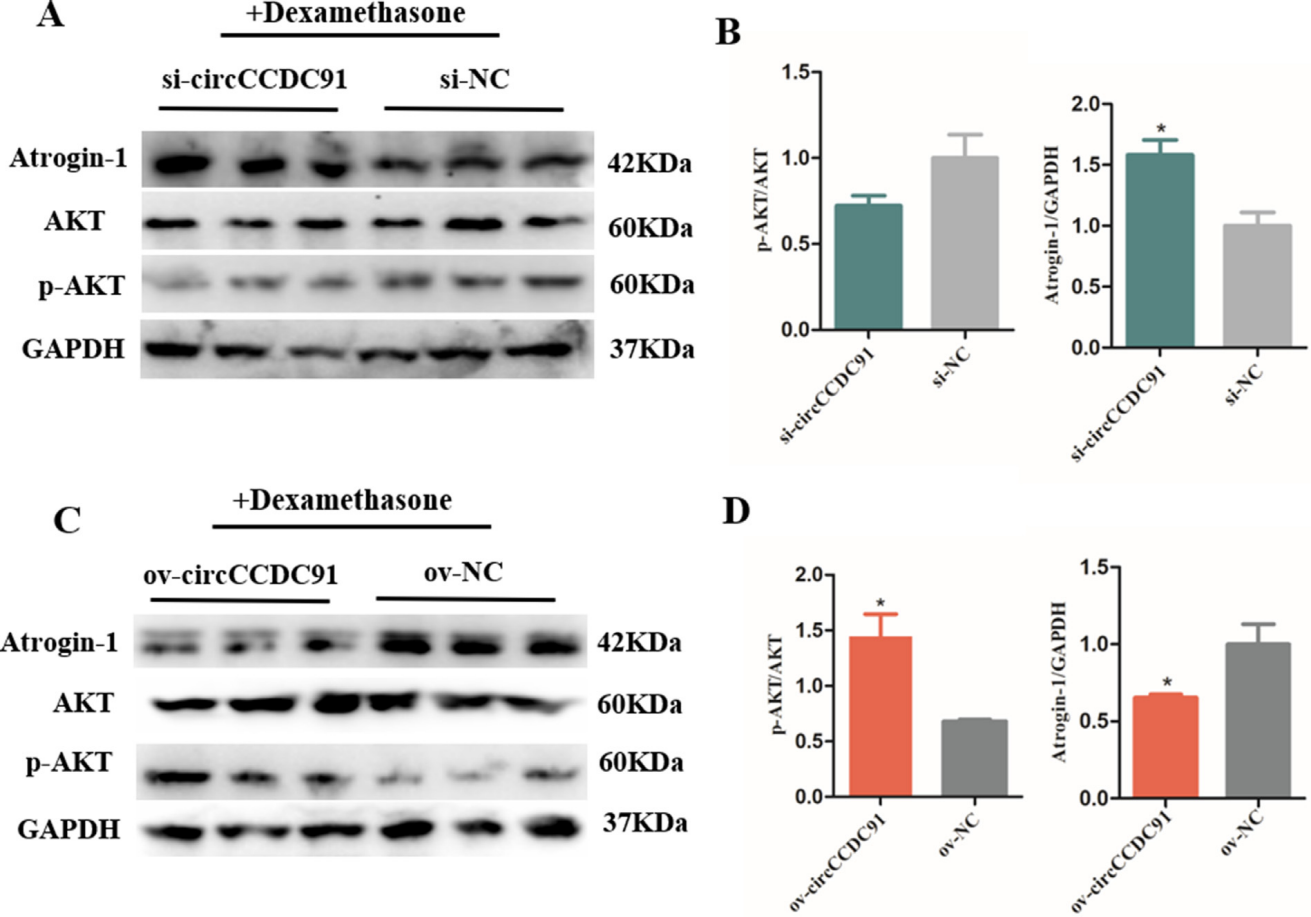
CircCCDC91 alleviates the effects of dexamethasone on muscle atrophy. (A) The western blot results of Atrogin-1, AKT, and p-AKT protein expression level of after treated with dexamethasone for 24 h, and followed circCCDC91 knockdown on myoblasts. (B) The quantification of Atrogin-1 and p-AKT protein expression level of after treated with dexamethasone for 24h, and followed circCCDC91 knockdown on myoblasts. (C) The western blot results of Atrogin-1, AKT, and p-AKT protein expression level of after treated with dexamethasone for 24 h, and followed circCCDC91 overexpression on myoblasts. (D) The quantification of Atrogin-1 and p-AKT protein expression level of after treated with dexamethasone for 24 h, and followed circCCDC91 overexpression on myoblasts. GAPDH serves as the internal reference for the experiment. Data are represented mean ± SEM. Each transfection group has three independent replicates. * *P* < 0.05; ** *P* < 0.01.

Previous reports showed that dexamethasone promoted protein degradation and increased the expression of Atrogin-1 and MuRF1, leading to skeletal muscle atrophy (Wang et al., 1998; Bodine et al., 2001a). For instance, Lee et al. (2018) investigated the function of Pyropia yezoensis Protein by inducing mouse skeletal muscle atrophy with dexamethasone in vitro. Moreover, Li et al. (2019) also explored the potential effect of lncIRS1 on skeletal muscle by treating chicken myoblasts with dexamethasone in vitro. In our study, we determined whether circCCDC91 could rescue muscle atrophy via activating IGF1-PI3K/AKT signaling pathway, we induced muscle atrophy with dexamethasone in vitro. Subsequently, we examined the protein levels of AKT, p-AKT, and Atrogin-1 in circCCDC91 interfered and overexpressed cells. Results showed that after the dexamethasone treatment, the expression of Atrogin-1 in circCCDC91 interference group were obviously higher than those in control group and the relative level of p-AKT was decreased after the circCCDC91 interference (Figures 7A and 7B). However, we found that the overexpression of circCCDC91 significantly alleviated the effects of the dexamethasone (Figures 7C and 7D). These results suggested that circCCDC91 could rescue skeletal muscle atrophy through IGF1-PI3K/AKT signaling pathway. In summary, we found that different circRNAs have diverse functions and mechanisms for numerous biological processes. This study provides a basis for subsequent research on the function of circRNAs and enriches the library of circRNAs. In addition, further study is required to discover more muscle development related circRNAs, in order to provide the basis for further molecular genetic studies to promote chicken muscle production and improving meat traits in chicken.

## CONCLUSIONS

**Figure 8:**
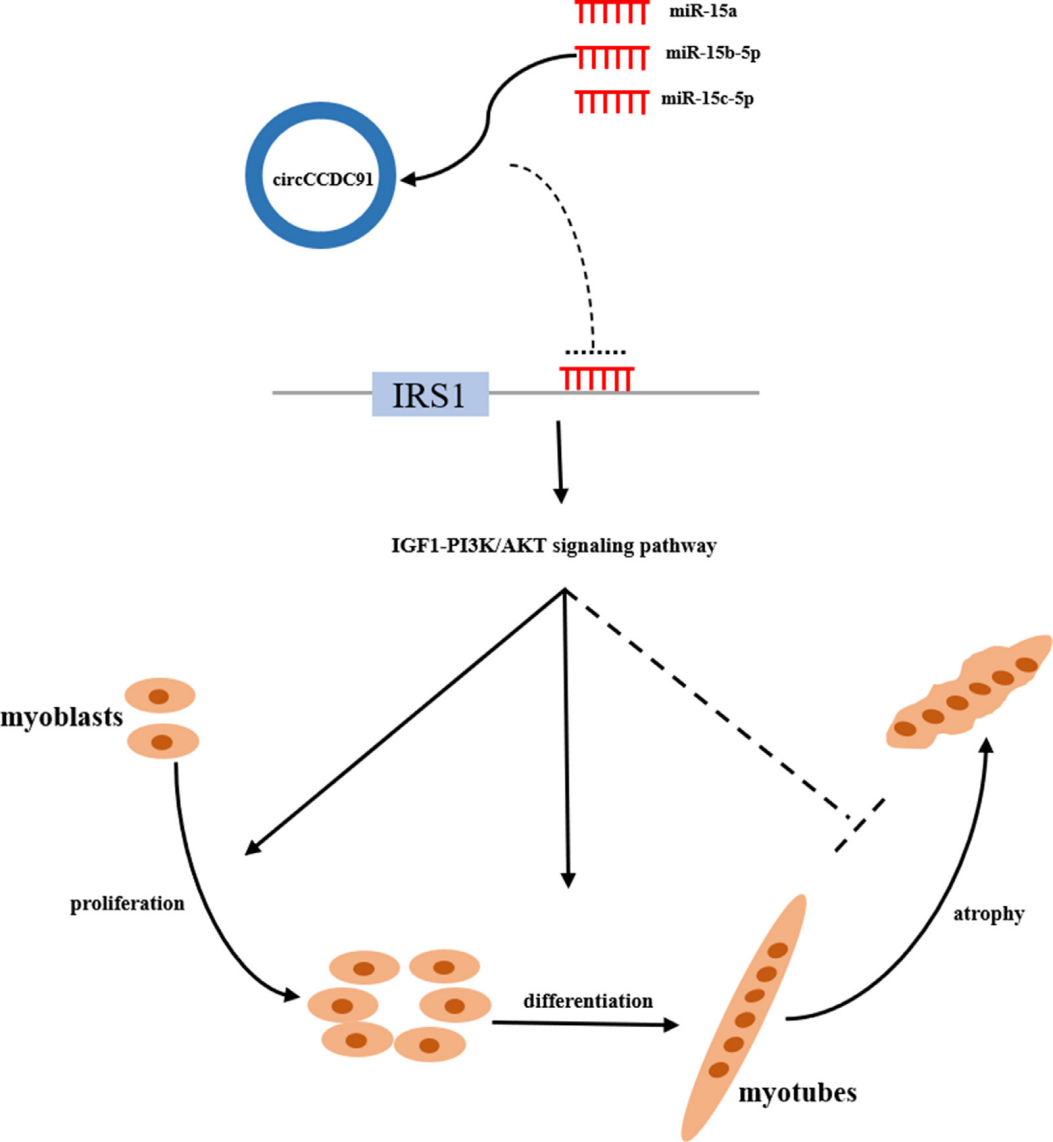
Mechanism of circCCDC91 regulates chicken skeletal muscle development by sponging miR-15 family via activating IGF1-PI3K/AKT signaling pathway.

In summary, our study revealed that a novel circRNA circCCDC91 promotes the proliferation and differentiation of chicken myoblasts and rescues muscle atrophy by suppressing the expression of miR-15a, miR-15b-5p, and miR-15c-5p via activating IGF1-PI3K/AKT signaling pathway (Figure 8).

## Appendix Supplementary materials

Image, application 1

Image, application 2

## Footnotes

1 All animal experimental procedures in this study were approved by the Animal Welfare Committee of Sichuan Agriculture University, and the assurance number is 2019-007.

Supplementary material associated with this article can be found, in the online version, at doi:10.1016/j.psj.2022.101803.
