# Supplementary material for: CircCCDC91 regulates chicken skeletal muscle development by sponging miR-15 family via activating IGF1-PI3K/AKT signaling pathway
Source: Poult Sci. 2022 Feb 25;101(5):101803. doi: 10.1016/j.psj.2022.101803 (PMC8956820; doi:10.1016/j.psj.2022.101803)
Supplement: Supplementary file 1 [file mmc1.docx]

|  | **Table S1** **Primers for qPCR** |  |
| --- | --- | --- |
| **Gene** | **Primer sequences (5'→3')** | **Application** |
| divergent primers | F: TACAGTTCAACAGCAAGAAG | validation of circCCDC91 |
|  | R: ATGGAATAGCAGGAGATGTC |  |
| circCCDC91 | F: TGAAGCCTTGAGTATTATTG | qRT-PCR |
|  | R: TACGGTAGAAATCCTGAAC |  |
| Myf5 | F: CCTCATGTGGGCTTGCAAA | qRT-PCR |
|  | R: CCTTCCGCCGGTCCAT |  |
| MyoD | F: GCCGCCGATGACTTCTATGA | qRT-PCR |
|  | R: CAGGTCCTCGAAGAAGTGCAT |  |
| MyHC | F: GAAGGAGACCTCAACGAGATGG | qRT-PCR |
|  | R: ATTCAGGTGTCCCAAGTCATCC |  |
| MyoG | F: CGTGTGCCACAGCCAATG | qRT-PCR |
|  | R: CCGCCGGAGAGAGACCTT |  |
| Atrogin-1 | F: TCAACGGGTCGGCAAGTCT | qRT-PCR |
|  | R: TCCCTCCCATCGCTCAGTC |  |
| MuRF1 | F: GGCAGCAGCATCATCTCGG | qRT-PCR |
|  | R: CCTCGCAGGTGACGCAGTAG |  |
| IRS1 | F: CTACCGCCTGTGCCTGACTAAC  R: CGTCCCACCTCGATGAAGAAG | qRT-PCR |
| GAPDH | F: CCAGAACATCATCCCAGCGTC | qRT-PCR |
|  | R: ACGGCAGGTCAGGTCAACAA |  |
| β-actin | F: GTCCACCGCAAATGCTTCTAA | qRT-PCR |
|  | R: TGCGCATTTATGGGTTTTGTT |  |
| miR-15a | F: GGGGGGTAGCAGCACATA | qRT-PCR |
|  | R: CAGGTCCAGTTTTTTTTTTTTTT |  |
| miR-15b-5p | F: TAGCAGCACATCATGGTTTGCA | qRT-PCR |
|  | R: CAGGTCCAGTTTTTTTTTTTTTT |  |
| miR-15c-5p | F: TAGCAGCACATCATGGTTTGTA | qRT-PCR |
|  | R: CAGGTCCAGTTTTTTTTTTTTTT |  |
| U6 | F: TGGAACGCTTCACGAATTTGCG | qRT-PCR |
|  | R: CAGGTCCAGTTTTTTTTTTTTTT |  |

| **Table S2** **the sequence of circCCDC91 and** **cyclization element (The blue represents cyclization element)**  TTTGTGCTGACAGCTAGGAATAAATGGGAAAAGCAGGAAACATATTTGATTTAATCACACTGCTGATTTGGAAAAACAACTTAAGAATAGACGCTGTTTTTATATTTTAATTTGTGATCACAGAGCTGTAAAGTACACTGCATATTTCCATATCATTAAATTGAGTTATGTTCATTAGTTATTAAGAAAATCCTTCATTTCAGAATCTAATGGTCTGAAATTCTGTTTTGTTTTGTTTTTAATATTCAGATGAACTGGGTTATGTTGCTGTTAGTATCATTTATGTAGGTACTAGAGAATTGACTGCTGTGTTTCAGAGCAATAGACATAACTTTAAAATTAACTTTTCCTATTTTCCCATCTTAAAAAGCATTTGTAACTGTCTTATTTTGAAAAGTTCTTCAATATTGTTGTGCTTATCTTTTCTCTTGAAGATTTTCAAATTCCAACTTAAAAGTTCAACCTTTTTTACTTGTATCAATAAAATTTGCTCTTAAAAGAAAGCTTGCAGCTCTTGTAACCTCTGTGTGAAATTAAAAAAAAAAAATTCTATAGGTCTCTTGATCTTTAGTAGAACTGGTACATTAGGATGATTTCAGGTACCTTGTGGAGTTCAAGTTAAACATTGAAATGACTGAATCCTGTTTTCTGAATGAGAAGGTGATAAGGTTAGGAAGAGCACATAGATGAGCTCTTGTATCCAAGTGTTTATTCATCTGTCATTTAACTGAGTTACAATATAGGAGTCCATTTTTTTCTTTTGACTCCTAGTTGATCATAAAATTAACACATTTGTCTTATGAAACTGACCAATGAAATATAATTATTTTCCTGCAGTGTACTGCGTTGTATTACTTCATCTTCCTGGTCTTCAGGATTTAACTACTGTAAATAAGTTTGTCCTTTAAAATGAACAGTATAATTTTCATTTTACAAATTTTTATTTTTTACTTAAACGGGATATAAATAATTAGTGATTTTGGTTTTGTTTTTAAATCAGGATTTCTACCGTACTGTAGTATTTGTTGAAAAATGGATGATGATGATTTTGGAGGATTTGAGGCAGCAGAGAGCTATGAGTGTGGAAATGGTGACAAGCAGATGACATCTCCTGCTATTCCATGGGCAGCATTTCCTACAGAGTTTGAAGTCCATATATCTCAGAATGTTTCTCCCAATGTTCTTCTGGAGCACTGTGTGCCTTCATCCTTCCTGGATGCTTCTGACTCATTCACTTCATCAAGTGATAATGTTGCAACATCTATTCAGAAAGTCAACAATGTGATAAACTCAGCTGTTCTTGAAGAACAGATTCAAGTAGATATTCCAGTTGCCTCTTTGAATTTAACAGAAGATAAGTCTTTAGTCACATCATCCGTTGCTATGGATGATGCTCAGACACAGAGAACTGATGAATCAAAGAGCTGCCTTCAACAAACTCTAGCAAATCTAGAAATCAATCTTTGTGCTGCTGAAGAAGAAAAATTAAAAATTAAAAAGGAATTGGAATATTTACTTAAAAAGCATAGTGTTCAAGAAATGGATTTTTTGAAGGAGAAAAAAGAAAAAGCTCTTTCACATGAAGATCATTACAAGATACTCCAGGAAAAGCATAAGCAGGAGTTAGAAGATATGAGAAAAGCTGGACATGAAGCCTTGAGTATTATTGTTGAAGAGTTCAAGTCATTGCTACAGTGTACAGTTCAACAGCAAGAAGCAGCTACTGAAAAACAGTATATACTAGCAATTGAAAAACATTCTTACAAATGTCAAGAGCTTCTTGATGTTCAGGTATGTATGTGCATTTACAAAAACCTTTATGATTGTCTTTTAGTTTCAGCTCTTTTTTTTGTTTGAAAAAAATATGCCTTTTGGAGCAAAGAGGATGACCAGTCTTTGATTTTGTGTCCTCAACATAACAGTAAACACAGATGATTAAAAATGCTAAGTGCATGGTAACTGTTTGATTTGAAAGTATTTTATAACACTTTTAAGACAAATGTGTTAATTTTATGATCAACTAGGAGTCAAAAGAAAAAAATGGACTCCTATATTGTAACTCAGTTAAATGACAGATGAATAAACACTTGGATACAAGAGCTCATCTATGTGCTCTTCCTAACCTTATCACCTTCTCATTCAGAAAACAGGATTCAGTCATTTCAATGTTTAACTTGAACTCCACAAGGTACCTGAAATCATCCTAATGTACCAGTTCTACTAAAGATCAAGAGACCTATAGAATTTTTTTTTTTTAATTTCACACAGAGGTTACAAGAGCTGCAAGCTTTCTTTTAAGAGCAAATTTTATTGATACAAGTAAAAAAGGTTGAACTTTTAAGTTGGAATTTGAAAATCTTCAAGAGAAAAGATAAGCACAACAATATTGAAGAACTTTTCAAAATAAGACAGTTACAAATGCTTTTTAAGATGGGAAAATAGGAAAAGTTAATTTTAAAGTTATGTCTATTGCTCTGAAACACAGCAGTCAATTCTCTAGTACCTACATAAATGATACTAACAGCAACATAACCCAGTTCATCTGAATATTAAAAACAAAACAAAACAGAATTTCAGACCATTAGATTCTGAAATGAAGGATTTTCTTAATAACTAATGAACATAACTCAATTTAATGATATGGAAATATGCAGTGTACTTTACAGCTCTGTGATCACAAATTAAAATATAAAAACAGCGTCTATTCTTAAGTTGTTTTTCCAAATCAGCAGTGTGATTAAATCAAATATGTTTCCTGCTTTTCCCATTTATTCCTAGCTGTCAGCACAAA  **Table S3 RNA oligonucleotides in this study** | |
| --- | --- |
| **Gene** | **Primer sequences (5'→3')** |
| s1-circCCDC91 | F: GAUACUCCAGGAUUUCUACTT |
|  | R: GUAGAAAUCCUGGAGUAUCTT |
| s2-circCCDC91 | F: ACUCCAGGAUUUCUACCGUTT |
|  | R: ACGGUAGAAAUCCUGGAGUTT |
| si-NC | F: UUCUCCGAACGUGUCACGUTT |
|  | R: ACGUGACACGUUCGGAGAATT |
| mimic NC | UUGUACUACACAAAAGUACUG |
| gga-miR-15a mimics | UAGCAGCACAUAAUGGUUUGU |
| gga-miR-15b-5p mimics | UAGCAGCACAUCAUCAUGGUUUGCA |
| gga-miR-15c-5p mimics | UAGCAGCACAUCAUGGUUUGUA |

**Figure S1. (A)** 4 circRNAs generated by the chicken CCDC91 gene. The green rectangles represent the exons of CCDC91. **(B)** The RNA-Seq result showed that the expression level of 4 circCCDC91s in E11, E13, E16 and E19. **(C)** The schema of circCCDC91 derived from the exon 2 to 8 of CCDC91. The red triangles represent divergent primers. Sanger sequencing confirmed the back-splicing junction sequence of circCCDC91. Red arrow points to the splicing site. **(D)** The qRT-PCR assay showed the resistance of circCCDC91 to RNase R digestion. **(E)** The circCCDC91 expression level was measured by qRT-PCR in different tissues and organs of chicken. In all panels, the values represent mean ± SEM from three independent experiments. * *P* < 0.05; ** *P* < 0.01.

**
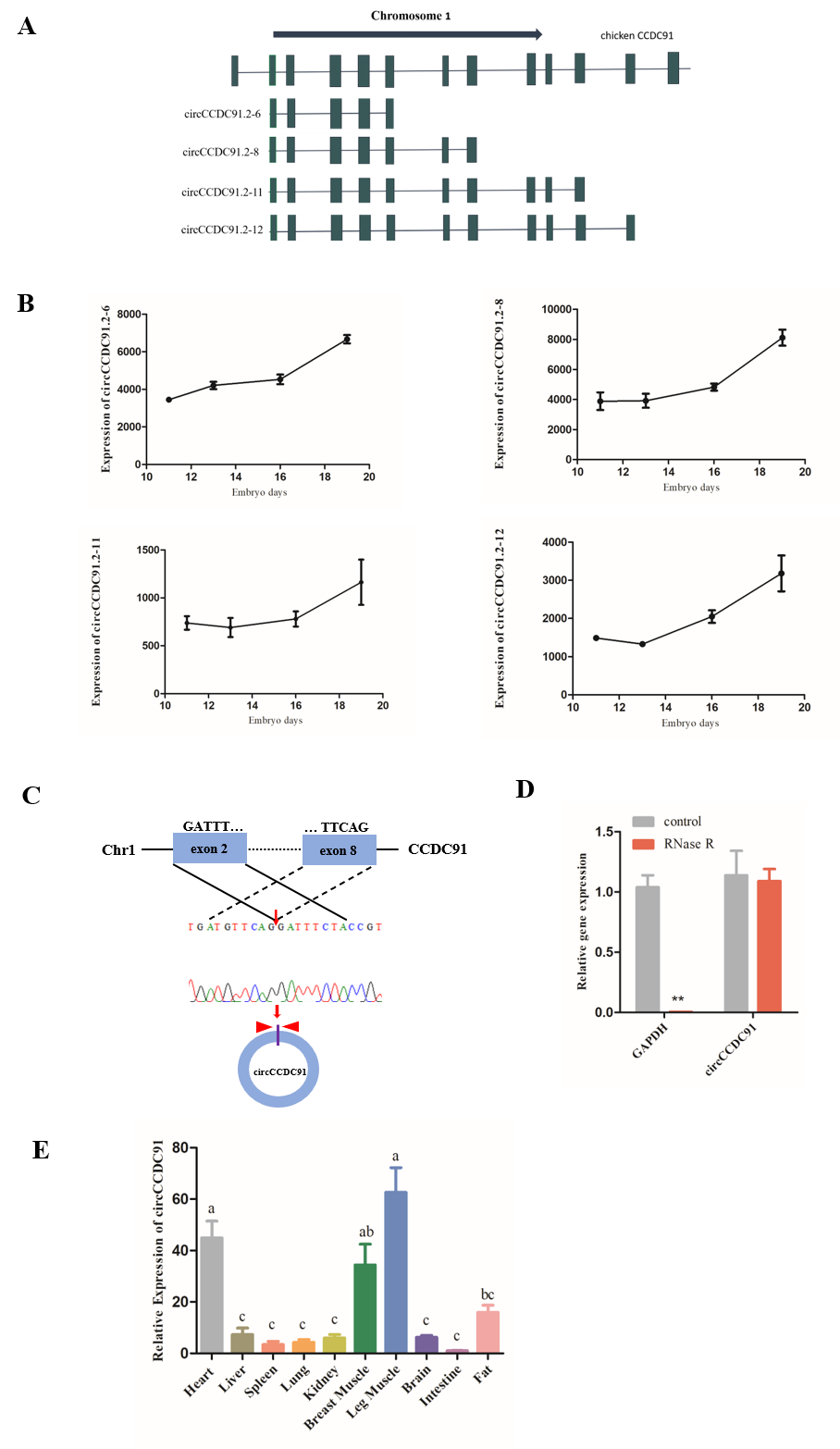
**
