# Supplementary material for: CircCCDC91 regulates chicken skeletal muscle development by sponging miR-15 family via activating IGF1-PI3K/AKT signaling pathway
Source: Poult Sci. 2022 Feb 25;101(5):101803. doi: 10.1016/j.psj.2022.101803 (PMC8956820; doi:10.1016/j.psj.2022.101803)
Supplement: Supplementary file 2 [file mmc2.docx]

**Full scans of immunoblots shown in the figure below.**


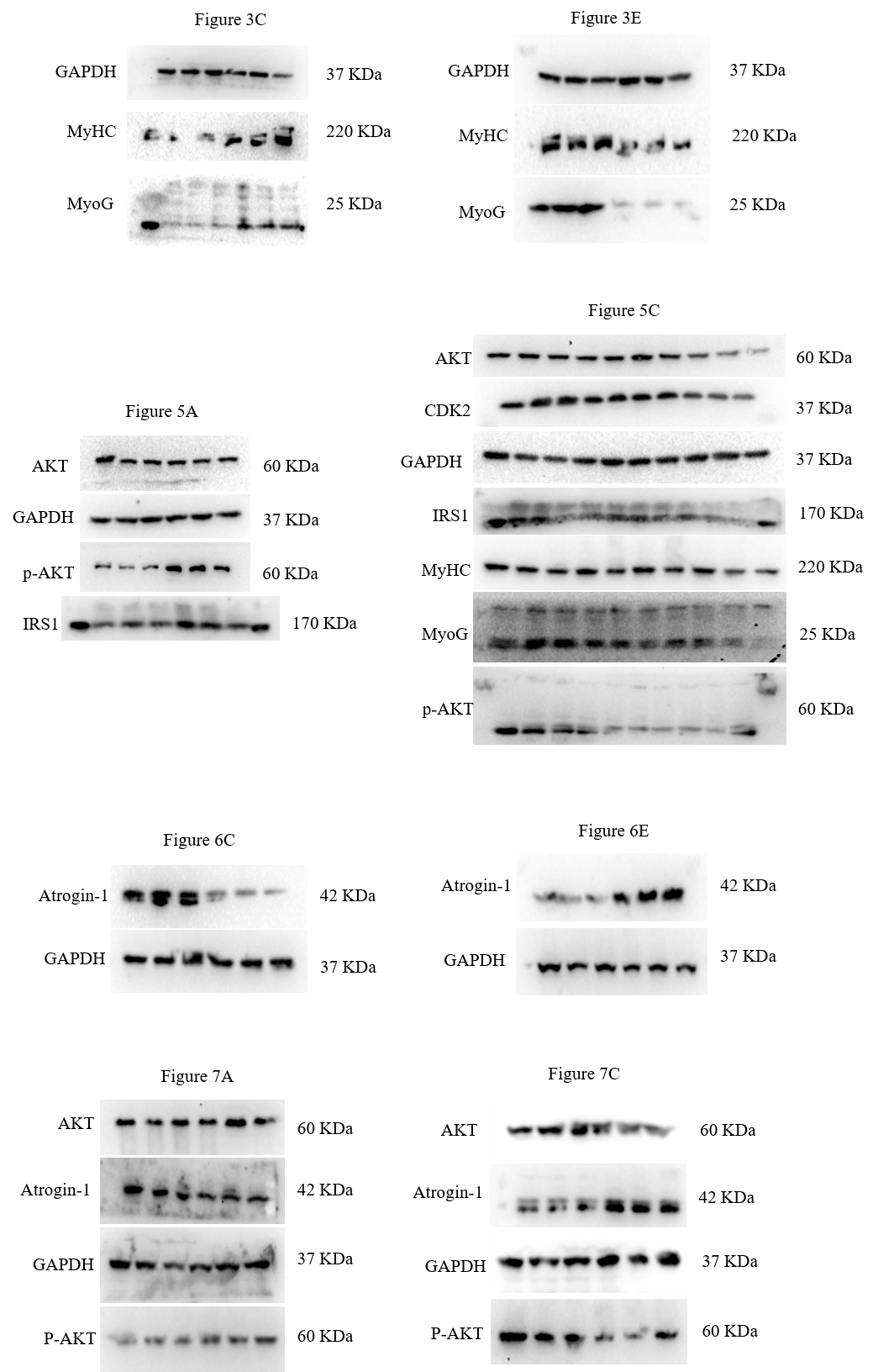


Original data for Figure 4F

| group | firefly luciferase | Renilla luciferase |
| --- | --- | --- |
| wt+nc | 9247000 | 244600 |
| wt+nc | 12720000 | 369000 |
| wt+nc | 10080000 | 277900 |
| mt+nc | 16290000 | 338800 |
| mt+nc | 18280000 | 489900 |
| mt+nc | 14860000 | 384100 |
| wt+15a | 573600 | 10330 |
| wt+15a | 1412000 | 32180 |
| wt+15a | 735200 | 15100 |
| mt+15a | 8469000 | 115000 |
| mt+15a | 13000000 | 200900 |
| mt+15a | 9264000 | 141200 |
| wt+15b | 4945000 | 209700 |
| wt+15b | 6202000 | 270200 |
| wt+15b | 4690000 | 193300 |
| mt+15b | 6572000 | 163900 |
| mt+15b | 8767000 | 228300 |
| mt+15b | 6543000 | 152100 |
| wt+15c | 5543000 | 193100 |
| wt+15c | 9570000 | 469100 |
| wt+15c | 5626000 | 202800 |
| mt+15c | 2102000 | 43500 |
| mt+15c | 3510000 | 76540 |
| mt+15c | 1568000 | 37910 |
